# Wide Awake Parenting: study protocol for a randomised controlled trial of a parenting program for the management of post-partum fatigue

**DOI:** 10.1186/1471-2458-13-26

**Published:** 2013-01-11

**Authors:** Melissa Dunning, Monique Seymour, Amanda Cooklin, Rebecca Giallo

**Affiliations:** 1 Parenting Research Centre, 5/232 Victoria Parade, East Melbourne, VIC, 3002, Australia

**Keywords:** Fatigue, Parents, Post-partum, Pycho-educational Intervention

## Abstract

**Background:**

Exhaustion and fatigue are commonly experienced by parents during the post-partum period, and can have implications for daily functioning, mental health and parenting practices. There is a need for the development of effective interventions to assist parents with the management of fatigue. This paper outlines the procedure for a randomised controlled study which aims to test the efficacy of *Wide Awake Parenting*, a program for the management of fatigue in the postnatal period.

**Methods/design:**

Parents with an infant less than 6 months of age, and from seven Local Government Areas in Melbourne, Australia were invited to participate in this study. Parents were randomised to receive the *Wide Awake Parenting* program (intervention groups) or usual care (control group) offered by health services. The *Wide Awake Parenting* program provides parents with psycho-education and information about fatigue, and strategies to reduce its effects either via a self-directed method, or professionally led with a home visit and telephone support. Baseline data will be collected prior to randomisation, and further data will be collected at 2- and 6-weeks post intervention.

**Discussion:**

To our knowledge this is the first randomised controlled trial of a program which compares the efficacy of a self-management approach and health professional assistance for the management of fatigue in the early post-partum period. If effective, it could offer an important, universal public health management approach to this common health concern.

**Trial registration number:**

Australian New Zealand Clinical Trials Registry, ACTRN12611000133932.

## Background

The experience of fatigue and exhaustion is a common health concern for parents, particularly those with young infants [1,2]. Emerging research suggests that fatigue is associated with parent mental health and wellbeing difficulties [3-5], as well as a range of adverse parenting behaviours [3,6,7]. Given that fatigue may have implications for parents’ functioning, there is a need for interventions to support parents to manage experiences of fatigue and exhaustion during the post-partum period. This paper presents the study protocol of a randomised controlled trial of a universal parenting program, *Wide Awake Parenting (WAP)* designed to assist with the management of fatigue and improve parent wellbeing.

The terms tiredness, fatigue and exhaustion are often used interchangeably by parents, and in the research literature. While tiredness is transient, fatigue refers to persistent exhaustion and lack of energy that is not easily relieved by rest or sleep [8]. For the purpose of this paper, the terms fatigue and exhaustion will be used interchangeably. In a qualitative study by Herbert [9], fatigue was found to be a significant health concern that emerged during the first few days following birth and remained at 3-months post-partum. Additional quantitative studies have also found fatigue to be one of the most commonly reported health concerns during the early post-partum period [1,2,10,11], and as many as 67% of Australian mothers report persistent tiredness at 6-months post-partum [12]. Although previous studies have typically focused on fatigue in mothers, emerging research highlights that fathers experience similar levels of fatigue as mothers [13,14] and that fatigue is a common experience for many fathers during the early parenting period [15].

There are a variety of reasons why parents of infants may become fatigued or exhausted. Pugh and Milligan [16] proposed a model of childbearing fatigue being influenced by physiological, psychological, and situational factors that may put a parent at risk of exhaustion and interfere with daily functioning. Research has found that birth-related factors such as breastfeeding [17,18] and caesarean delivery [19], as well as demographic and family characteristics including older maternal age [20,21], sole parenting [3,21] and low income [22] to be associated with fatigue in parents. Additionally, during late pregnancy and the post-partum period, parents experience significant changes in their sleep patterns, including less night-time sleep and more disrupted sleep, which may contribute to feelings of exhaustion [13,14,23]. Parents with infants who frequently wake at night are at risk of experiencing clinically significant levels of fatigue and exhaustion [24].

Furthermore, recent Australian research on fatigue in the early parenting period suggests that fatigue can last well beyond infancy [3]. This study found that a variety of socio-ecological factors place mothers and fathers at increased risk of experiencing fatigue, including poor sleep quality, insufficient social support, poor diet and exercise, ineffective coping styles, sleep quality and child temperament [3].

### Effects of fatigue on parent wellbeing and behaviour

The experience of fatigue, while often anticipated, is largely under-prepared for by new parents [9]. Fatigue not only has the potential to interfere with how parents adjust to their new role as a mother or father, but also for the resumption of previous roles and responsibilities, including those in paid work, social and family settings [1,16,25]. It is well documented that fatigue can impair daily functioning [24], social judgment and cognitive functioning, such as memory, decision-making, concentration and planning [26-29]. The same is true for parents. In a study of 109 Australian mothers attending a public mothercraft unit, mothers who were clinically exhausted were found to experience significant impairments in their daytime functioning and reduced clarity of thought [24].

Fatigue is likely to impact parental wellbeing. A study by Bayer et al. [30] found that maternal tiredness in a community sample of Australian mothers with infants (3-6months) was associated with poorer mental and physical health, including symptoms of depression, anxiety, stress and fatigue/energy. The array of extra demands placed on parents along with the experience of fatigue is likely to add to parental stress and decrease mood [31]. Although fatigue and loss of energy are part of the diagnostic criteria for clinical depression it has been found that the two are separate, distinct, unique psychological constructs [13,32-35]. Fatigue has been found to be strongly related to symptoms of depression and anxiety during the prenatal [1,36] and postnatal period [33,37,38]. It is likely that the two conditions exacerbate each other [21]; fatigue can heighten feelings of depression, anxiety and helplessness, which in turn leads to further exhaustion [36].

It is also likely that fatigue adversely affects parenting practices and experiences. Emerging research suggests that fatigue can increase parenting stress [3,6,39], limit patience in coping with infant crying [39], and decrease parenting confidence, satisfaction and self-efficacy [3,6,7]. Recent Australian research shows that mothers and fathers who are fatigued are using more irritable, frustrated and impatient parenting behaviours directed towards their child [3]. Studies also show that fatigued parents are less warm and are less likely to be involved with their child [3,7,40]. Not surprisingly, fatigue has the potential to impact upon parents’ ability to care for and interact with their infant or child [4,30].

Given that fatigue can adversely affect the ability to parent optimally, it also has the potential to influence child outcomes and development [41]. Parks et al. [2] found an association between mothers reporting persistent fatigue at 18 months post-partum and poorer eye-hand performance in their infants. While research in this area is limited, associations have also been found for other wellbeing difficulties. For example, Kahn, Zuckerman, Bauchner, Homer and Wise [42] found that depression in mothers was associated with poorer physical health and increased behaviour problems in their children at age three.

Fatigue plausibly presents a negative cycle for parents; parents who feel tired may not sustain self-care behaviours; they then may become ill, which in turn may influence their ability to get enough sleep and rest as they deal with daily demands; resulting in more fatigue [2]. Fatigue can also result in increased conflict between partners [30] and can have a detrimental impact upon family relationships and family functioning [25]. The relationships between parental fatigue, parenting and child development described above are also likely to be complex and bi-directional. Reducing or preventing one wellbeing difficulty, such as fatigue, may have a multitude of benefits on other aspects of a parent’s life.

### Current interventions and management of fatigue

With growing evidence suggesting that fatigue is a serious health and wellbeing concern for many parents, little research has been conducted on strategies to manage and reduce parental fatigue. Research identifies the need for psycho-education about the effects of fatigue and strategies to promote self-care behaviour to conserve energy, improve quality of diet, increase exercise, reduce daily demands, maintain regular sleep routines, and allowing time for rest [2,4,43]. Although a common experience for many new parents and a topic parents would like to discuss more [12], mothers have reported feeling that it is inappropriate to seek professional support for their fatigue and it is often minimised or overlooked by health professionals [10].

A randomised control study of a self-care intervention to reduce fatigue in a sample of 68 first-time mothers was conducted by Troy and Dalagas-Pelish [44]. Mothers were given a *Tiredness Management Guide* which described eight potential sources of post-partum fatigue, including infection, lack of daytime rest, pressure to get everything done, interruption in night-time sleep, pain, stress, anaemia and social activities. The guide listed several techniques for each source of fatigue and asked mothers to try some of the suggestions whenever they felt tired. Mothers completed the intervention over a four week period from two weeks post-partum to six weeks post-partum. Results revealed whilst the use of the self-directed intervention reduced morning fatigue at the fourth week post-partum, overall the intervention was not associated with a significant decrease in fatigue from the second week to the sixth week post-partum as compared to mothers in the control group.

A more promising intervention was trialed by Thome and Alder [33]. Their randomised control study investigated the use of a telephone intervention to reduce symptoms of fatigue. Seventy-six mothers of behaviourally difficult 2-3 month old infants participated in the study. The intervention commenced when infants were 4-6months, lasting approximately two months in duration, where each mother received five telephone calls from a registered nurse. The intervention was based on cognitive-behavioural techniques and information and advice was given on topics related to maternal distress (e.g., fatigue, health problems, role conflict) and infant behaviours (e.g., sleep, crying, feeding and health problems). Results revealed that mothers who received the telephone intervention had a significant reduction in fatigue over time than those mothers in the control condition. This intervention may prove to be effective in managing symptoms of fatigue in parents as they receive one-on-one support from a trained health professional, allowing them a context to discuss and obtain additional information and strategies that are tailored to their specific needs. The results from the control group show that if nothing was done to relive maternal fatigue, it did not decrease overtime, suggesting that mothers need specific and targeted interventions to combat fatigue. Whilst this appears to be evidence for the treatment of fatigue, mothers in this sample were approached to participate as they had reported having a difficult baby in a previous study, these findings may not be generalizable to a community sample of parents.

Taken together the findings from the existing interventions above suggest that a parent-led, self-directed intervention may not be effective in preventing or managing post-partum fatigue. There is promising evidence however, that short-term, direct support from a health care professional may be beneficial in reducing the symptoms of parental fatigue.

### Development of Wide Awake Parenting

Research suggests that reducing daily demands, improving quality of diet and exercise, maintaining a regular sleep routine, promoting realistic parenting expectations, and encouraging help-seeking, can be useful for the management of fatigue [2,4,43]. Similar strategies were reported to be beneficial by parents of young children aged 0-6 years attending focus groups [45,46]. WAP was developed as a psycho-educational program based on evidence about factors associated with fatigue and strategies for the management of fatigue, as outlined above.

A preliminary study evaluating the content of WAP was conducted with 49 parents of young children aged 0-4 years attending a residential program for sleep and settling problems [47]. The program was delivered in the form of brief 90-minute workshop that provided psycho-education about fatigue and the potential impact of fatigue on parenting and wellbeing. Practical strategies to recharge and save energy were offered. These included taking time out for self, prioritising, problem solving, getting support, challenging unhelpful thinking, diet and exercise, and sleep hygiene. The program was found to have short-term effects on strengthening parents’ self-efficacy, their intention to engage in self-care behaviours, parents’ beliefs about the importance of self-care and perceived expectations of the benefits from managing fatigue. In addition to the short-term effects, the program was found to be acceptable and relevant to the needs of parents. The results of this pilot therefore provide encouraging support for the use of a psycho-educational based program for the management of parent fatigue in the post-partum period.

The aim of this paper is to present the study protocol for a randomised controlled trial of a psycho-educational intervention, WAP. The aim of WAP is to reduce fatigue, and promote parents’ well-being during the early post-partum period in a self-selected, community sample of parents. The content and development of WAP has been published elsewhere [47]. The recruitment phase of this trial is currently complete, with intervention delivery and data collection ongoing.

## Method/design

### Study design

Randomised controlled trial.

### Ethics approval

Approvals were obtained from the Parenting Research Centre Human Research Ethics Committee and the Department of Early Education and Early Childhood Development, Early Childhood Research Committee.

### Setting

Seven Melbourne (state of Victoria, Australia) Local Government Areas (LGAs; Wyndham, Stonnington, Glen Eira, Darebin, Whitehorse, Greater Dandenong and Casey) were chosen for recruitment given the high birth rates per annum reported in 2008 [48]. Within each LGA, parents were recruited through Maternal and Child Health services (MCHNs) and early childhood immunisation sessions. MCHNs provide a universal, free service offered to all Victorian families with scheduled family visits (covering 93% of all births) from the post-partum and up to 42 months of age. MCHNs run a range of individual, family and group services to families with infants and pre-school aged children (<6 years of age), including “new parent” education groups for first-time parents, aimed at providing parenting information and support. Free, universally accessible early childhood immunisations are provided in Australia, under the National Immunisation Program, via MCHNs in regular centre-based sessions. In two of the participating LGA’s, immunisation services are provided by the Environmental Health Unit (Casey and Wyndham) and not the MCHNs.

### Participants

#### Inclusion criteria

Mothers and fathers of infants aged 0-6 months, seen by their MCHN, attending an immunisation session or “new parent” group in one of the seven participating LGAs were eligible to participate in the study.

At the routine visits, participating MCHNs provided eligible parents’ with a flyer containing information about the study so that they could contact the research team, or gained permission to pass on the parent’s contact details to the research team on parents behalf. The study team contacted interested parents to provide further verbal information about the study. Participant information and consent forms were sent to parents who were willing to participate, and for partnered parents, a separate information and consent form for partners was also sent.

At immunisation sessions and “new parent” groups, the research team approached parents to ascertain their eligibility and to provide them with verbal and written information about the research. Contact details for interested parents were obtained, and parents and their partners were provided hard copies of the participant information and consent forms, either by post, or in person.

#### Exclusion criteria

Parents were excluded from the study if they were under the age of 18 years, had a child over the age of six months at the time of being approached to participate, were living outside one of the seven LGAs or had insufficient English to complete the questionnaires.

### Sample size/power calculation

Power analysis using the program 'GPower 3.1.2' was conducted to determine the required sample size for the study. A 3-group comparative study would require a total sample of 184 participants (approximately 61 parents in each treatment condition) to have 80% power to detect a statistically significant finding at *p* < .05 and a moderate effect size for the short-term outcomes at two weeks post-intervention. Allowing for a 20% dropout rate, we aimed to recruit approximately 221 parents (approximately 74 participants in each treatment condition).

### Randomisation

Families were randomised upon receipt of their signed consent form and baseline questionnaire. Prior to the recruitment period, a computer generated random number sequence provided by http://www.random.org was established for each participating LGA. This allowed for each parent or family (if both parents participated) per LGA to have equal chance of being allocated to either of the intervention conditions (i.e., written information with telephone support or written information only) or control condition. The research team and families remained blind to group allocation at the time of recruitment and consent. However, following the return of the participants consent form, knowledge of group allocation is unavoidable given the type of intervention. Thus, each parent or family were allocated to one of the intervention or control conditions and notified of this allocation.

### Intervention

#### Written information condition

Parents allocated to the WAP written information group were sent the WAP intervention workbook. The 44-page workbook is broken into four parts designed to be used in a self-directed manner over a period of 4-weeks. The first part provides information about fatigue and exhaustion, including what it is, how it can affect parents, and why it occurs. The second part provides tips for charging up and includes strategies such as taking time out for self, helpful thinking, sleeping better and resting, eating well, and keeping active. The third part provides tips for saving energy and includes strategies such as prioritising, problem solving, and getting support. Throughout these sections of the workbook are activities to help parents think about ways to apply the strategies and make them relevant to their individual needs. The notion of developing a goal and plan to make it happen is also emphasized. The final part of the workbook then provides advice on how to make the plan work and contact information and websites for additional support services parents may choose to pursue.

#### Written information with telephone support condition

Participants in this arm received professionally-led, structured guidance to supplement the written information provided in the WAP booklet, over the four weeks of the programme. Parents received an initial home visit from a trained facilitator. The purpose of the home visit was to establish rapport with the parent, provide a WAP workbook, introduce the program and work through the first section of the workbook. At the home visit, three telephone sessions were scheduled at weekly intervals for the following three weeks, corresponding with one per session of the workbook. During the telephone sessions, the facilitator checked the parent’s understanding of the workbook content, clarified any information, answered questions, or guided progress through the material when necessary. Parents were encouraged to think about specific strategies relevant to their needs, to address barriers to these strategies, to develop goals, and ultimately, devise a plan to support them with the management of fatigue.

#### Waitlist control condition

Parents allocated to the waitlist control group initially received no intervention or resources from the research team. These families continued to receive the usual assistance and advice provided to all parents of newborns, via usual contact with MCHNs and other health professionals. Once the final follow-up questionnaire was received by the research team from parents in the waitlist control group, they were offered the WAP intervention and had the choice of receiving written information only or written information with telephone support.

### Measures

#### Demographic questionnaire

Information about parent age, gender, marital status, educational background, employment status, and demographic information about other children in the family were collected. The *Australian Bureau of Statistics, Socio-economic Indexes for Areas*[49] was used to identify families’ socioeconomic status based on their postal code. The Index of Relative Socioeconomic Disadvantage was used and is based on variables such as low income, low educational attainment, and high unemployment from the 2001 population census data. High scores reflect an area of relatively better economic status.

#### Primary study outcome

Primary study outcomes are parent-reported symptoms of fatigue, assessed at baseline and at short- (2 weeks) and longer-term follow-up (eight weeks).

*Fatigue Assessment Scale (FAS)*[50] is a ten item measure that will be used to assess the physical and cognitive symptoms of fatigue. The items are rated on a 5-point scale, ranging from 1 = *Never* to 5 = *Always*, with higher scores indicating higher levels of fatigue. The scale is reported to have good psychometric properties, and has been evaluated for use with parents [35].

*Fatigue Severity Scale*[51,52] assesses fatigue intensity and functional limitations caused by fatigue. The nine items (e.g., “Fatigue interferes with my work, family, or social life”) are rated on a 7-point scale, ranging from 1 = *Strongly Disagree* to 7 = *Strongly Agree*. Higher scores reflect higher levels of fatigue.

#### Secondary study outcomes

Secondary study outcomes, assessed at baseline and follow-up include parent-reported mental health, daily functioning, well-being and sleep quality; beliefs, intentions and engagement in self-care activities; parenting stress, sense of competence and parenting behaviours; and family functioning.

*Depression Anxiety and Stress Scale (DASS-21)*[53] assesses the negative emotional states of depression, anxiety and tension or stress over the past week. Items are rated on a 4-point scale ranging from 0 = *Does not apply to me at all* to 3 = *Does apply to me very much or most of the time*. Response are summed and multiplied by 2 to obtain a total score for each scale. Higher scores indicate greater symptoms on that scale; recommended cut-off scores for severity labels (normal, moderate, severe and extremely severe) are given in the DASS manual.

*Profile of Mental States (PoMS)*[54] is an adjective checklist of six mood factors, designed to measure mood variations over time in psychologically normal populations. Three subscales from the PoMS were used in this study: Anger – Hostility which assesses irritability (12-items); Confusion-Bewilderment assesses clarity of thought (7-items); and Vigour-Activity assesses energy and alertness (8-items). Participants rate their responses to each of the adjective items on these subscales on a 5-point scale and responses are summed to give a total score for each of the three subscales. Community norms for the PoMS subscales are available, and the PoMS has been widely used in research around Australian community samples during early parenting.

*Pittsburgh Sleep Quality Index (PSQI)*[55] is a standardised assessment of the overall quality of sleep. Two subscales from the PSQI were used in this study to assess participants’ sleep quality: a global rating of overall sleep quality, and the two item assessment of ‘daytime dysfunction’, assessing the degree to which fatigue impacts upon routine daily function and tasks. Two additional items have been added to ask parents about the reason for sleep disruption. Items are rated on a 4 point scale (0 = *Not during the past month* to 3 = *Three or more time a week*), where higher scores indicate poorer sleep quality.

*Beliefs about Self-Care Survey*[47] was designed specifically for this project, and is based on Ajzen and Fisbein’s Theory of Planned Behaviour/Reasoned Action. The items assess parents’ attitude toward self-care behaviour (10-items); peer and social norms influencing their thoughts about the self-care behaviour (4-items); and identifying barriers to engaging in self-care behaviour (10-items). Participants rate their response to each of the survey items on a 7-point scale, ranging from 7 = *Strongly Agree* to 1 = *Strongly Disagree*.

*Health and Self-care Behaviour*[47] consists of three items which ask respondents to rate the quality of their diet, their overall level of physical activity and the extent to which they engage in self-care behaviours such as relaxation, taking time out, and pursing hobbies on a 7-point Likert scale, ranging from 1 = *Strongly Disagree* to 7 = *Strongly Agree*.

*Parenting Sense of Competence Scale (PSOC)*[56] is a 16-item self-report measure that was used to assess parents’ satisfaction and efficacy in their parenting role. The Satisfaction subscale assesses parental motivation versus parenting frustration and anxiety. Items are rated on a 6-point Likert scale (1 = *strongly agree* to 6 = *strongly disagree)*. High scores on both subscales indicate high degrees of satisfaction and efficacy in parenting.

*The Parenting Stress Index (PSI)*[57] is a widely-used self-report assessment of the total stress parents experience within the daily interactions of parenting in the early years. Only the Difficult Child and Parent–child Dysfunction subscales were used; the Difficult-Child subscale reflects sources of parental stress associated with child characteristics that make it difficult to fulfill the parenting role, while the Parent–child Dysfunction subscale reflects parental stress associated with dysfunction in the parent–child relationship. Each subscale consists of two items, rated on a 5-point Likert scale (1 = *strongly agree* to 5 = *strongly disagree*). Higher scores indicate higher stress in the respondents role as a parent, while lower scores may be related to low stress, or dysfunctional parent–child relationships.

*Parenting Practices - Hostility and Warmth.* These items were taken from the Growing Up in Australia: Longitudinal Study of Australia Children [58]. Parental warmth refers to expressions of affection, showing interest and enjoyment in the child’s interests and activities; while Parenting hostility refers to anger and losing temper with the child. These parenting behaviours are of particular interest in the current study as they have been linked to a broad range of psychosocial, developmental and behavioural outcomes for children [59-63]. Participants rate 11-items (warmth, 6-items; hostility 5-items) on a 5-point Likert scale, ranging from 1 = *Never/Almost Never* to 5 = *Always/Almost Always*. Higher scores on each subscale indicate higher parental warmth and greater parental hostility.

The *Family Assessment Device (FAD)*[64]*.* The General Functioning scale from the FAD was used in the current study. This assesses the overall health and function of the family. The 12-items are rated on a 4-point scale, ranging from 1 = *Strongly Agree* to 4 = *Strongly Disagree*. The higher the score the more problematic the participant perceives their family’s overall functioning.

*Resources and support.* This part of the survey asked parents to indicate which support services or resources they have accessed since their baby was born. This is asked because we would like to be able to control or account for the types of support parents have accessed. Furthermore, it allows us to address in analyses any ‘contamination’ in the waitlist control group via exposure to WAP resources.

#### Satisfaction survey

Upon completing the intervention, participants in the written information and written information with telephone support groups were given a ‘satisfaction survey’ to evaluate the program. The satisfaction survey comprised of 14-items, which assessed the relevance and satisfaction with the program content and delivery method on a 5-point scale ranging from 1 = *Strongly Disagree* to 5 = *Strongly Agree*. Additionally, there were two open-ended items that allowed parents to discuss what they liked most about the program and ideas on how to improve the program.

### Procedures

Following initial contact, interested parents were assigned a participant identification code to protect confidentiality of all data provided, and an information pack. The pack contained a plain language statement, consent form, and the baseline questionnaire. If the parent was willing to participate they were able to return the hardcopy consent form and baseline questionnaire via reply paid envelope or could complete it online via a secure, web-based survey provider, indicating written consent on the form provided in the electronic survey. Two reminder calls, two weeks apart, were placed to parents who had not returned their baseline information. Those who did not return data following these calls were considered to have declined to participate.

Once the consent form and baseline surveys were received, participants were randomly allocated to one of the study groups, and were notified of which group they were randomised to. If the participant was allocated to the written information with telephone support group, a home visit was scheduled during this telephone call. If the participant was allocated to the written information only group, the intervention resources were posted out immediately after the telephone call. Participants who were randomised to the waitlist group were informed that they would not have access to the study intervention straight away but have the option of receiving either the written information only or the written information with telephone support upon receiving their final questionnaire in three months’ time.

Follow-up questionnaires were posted out to participants at two weeks, and six weeks following completion of the intervention for those in the intervention arms, and at six and 12 weeks for those in the control arm. Participants in either intervention arm were prompted to complete the satisfaction survey either during their last telephone session, or via phone, one month following provision of the WAP workbook, for those in the written only condition. In order to minimize study drop-out, researchers called participants if they had not returned their follow-up questionnaires at two and four weeks after they were mailed out to them. Control participants were advised that they would be contacted once their final survey was received and would be offered the WAP intervention.

### Hypotheses

Firstly, we hypothesised that compared to a waitlist control condition, parents receiving either form of the intervention will report the following short-term outcomes at 2-weeks post intervention:

a) Stronger beliefs and intention about engaging in self-care behaviour.

b) Higher levels of self-care behaviour.

c) Lower levels of fatigue and daytime dysfunction.

d) Lower levels of irritability, improved vigour and clarity of thought.

e) Improved sleep quality.

f) Lower levels of parenting stress and parenting hostility, and higher levels of parental warmth.

Secondly, we hypothesised that compared to a waitlist control condition, parents receiving either form of the intervention will report the following longer-term outcomes at 6-weeks post intervention:

a) Higher levels of parental self-efficacy.

b) Lower levels of depression, stress and anxiety.

c) Higher quality of the couple relationship and parent–child interactions.

Finally, we hypothesised that compared to the written information only condition, the intervention effects will be significantly stronger for parents in the written information with telephone support condition.

### Data analysis

A range of descriptive and multivariate data analyses will be used to assess the aims of the study. Descriptive statistics will be presented of the demographic characteristics of the sample. Means and standard deviations will be given for continuous outcomes, as well as medians and inter-quartile ranges where continuous data are skewed. Frequencies for categorical data will be reported. A mixed-design analysis of variance (split-plot ANOVA) will be conducted to compare the intervention and control conditions (between-subjects factor) on each of the primary and secondary outcomes at pre, post and follow-up (within-subjects factor). Effect sizes will be reported where appropriate, with 0.01, 0.06 and 0.14 as small, medium and large effect sizes for multivariate *η*^2^, while 0.2, 0.5 and 0.8 are small, medium, and large effect sizes for Cohen’s *d.* Finally, intention-to-treat analysis will be conducted using the last observation carried forward method for the missing post-intervention and follow-up data.

## Competing interests

The authors declare that they have no competing interests.

## Authors’ contributions

MD, MS, AC and RG all contributed to drafting and revising the manuscript. AC and RG are responsible for the study design. All authors read and approved the final manuscript.

## Pre-publication history

The pre-publication history for this paper can be accessed here:

http://www.biomedcentral.com/1471-2458/13/26/prepub
